# Cooling a Rotating Mirror Coupled to a Single Laguerre–Gaussian Cavity Mode Using Parametric Interactions

**DOI:** 10.3390/nano12203701

**Published:** 2022-10-21

**Authors:** Qiaoyun Pan, Weiyu Lv, Li Deng, Sumei Huang, Aixi Chen

**Affiliations:** School of Science, Zhejiang Sci-Tech University, Hangzhou 310018, China

**Keywords:** cavity optomechanical, ground state cooling, optical parametric amplifier

## Abstract

We study the cooling of a rotating mirror coupled to a Laguerre–Gaussian (L–G) cavity mode, which is assisted by an optical parametric amplifier (OPA). It is shown that the presence of the OPA can significantly lower the temperature of the rotating mirror, which is very critical in the application of quantum physics. We also find that the increase in angular momentum has an influence on the cooling of the rotating mirror. Our results may provide a potential application in the determination of the orbital angular momentum of light fields and precision measurement.

## 1. Introduction

In recent years, cavity optomechanical systems have become a popular research field in quantum optics, which focus on the interaction between a mechanical oscillator and light field via radiation pressure. This kind of interaction makes the optical degrees of freedom and mechanical degrees of freedom couple and let the cavity optomechanical system play a role in precision measurement and force sensors [1,2,3]. When the cavity optomechanical system is driven by a strong coupling field, it also becomes transparent to the input probing field due to the destructive interferences between the input probing field and the anti-Stokes fields generated by the interactions of the coupling field with the cavity, which is called optomechanically induced transparency (OMIT) [4,5,6]. In the OMIT, quantum coherent manipulation takes an important role in modulating the output light field, which is very similar to electromagnetically induced transparency (EIT) [7,8,9]. At present, OMIT has become a coherent control technology and is extended to studying optomechanical system coupling with other media [10,11,12,13,14,15,16,17,18]: Zhang and his coworkers proposed an atom-assisted cavity optomechanical system, consisting of a single Λ-type three-level atom, where steady-state solution of electromagnetically and optomechanically induced transparency and amplification is presented [10]. Based on the technology of OMIT, Xiao’s group investigated optomechanically induced entanglement in the conventional single-cavity optomechanical system coupling with a relatively weak probe field and a strong control field [11], and Li and his coworkers proposed to realize cooling of a mechanical resonator through an OMIT-like cooling mechanism in a double-cavity optomechanical system [12]. In addition, atomic ensemble, Kerr medium, optical parametric amplifier (OPA), and so on are introduced in the optomechanical system. These hybrid optomechanical systems can show strong nonlinearity effects under the condition of coupling of optical fields, so researchers can achieve steady-state entanglement [13,14], photon blockade [15], and the cooling of a mechanical oscillator [16,17] in hybrid systems.

About the cavity optomechanical system, some physical phenomena mentioned above are mainly based on the interaction between mechanical oscillator and light field via radiation pressure originating from momentum effects from the light field. For the light field, the mechanical effects of angular momentum are also worthy of research and analysis and some research works have paid attention to effects of angular momentum of the light field. When considering the mater interacting with the light beams with orbital angular momentum, nonlinear behavior and frequency shifts in the medium arising from their rotation are reported [19,20]. Explicit analytical expressions for the first few non-zero moments of the beam orbital angular momentum are obtained and potential applications in inertial confinement fusion, laser micromachining, and imaging are discussed [21]. Allen and Barnett have made many analyses on Laguerre–Gaussian (L–G) light mode with good angular momentum [22,23]. Based on the L–G light mode, optomechanically induced transparency is investigated in Ref. [24]. L–G optical sum-sideband generation via orbital angular momentum exchange is studied in an optorotational system [25]. 

Due to Laguerre–Gaussian spiral phase systems with remarkable optorotational interaction, many theoretical and experimental studies have begun to focus on this field recently and it has been an emerging subject and led to many applications, including sideband generation [25], cooling of rotational mirrors [26], and entanglements [27,28]. In the L–G optorotational system, the rotating mirror can be seen as a mechanical torsional pendulum. The theoretical studies show that the Hamiltonian of the L–G optorotational system is similar to that of the cavity optomechanical system. Based on this idea, we investigate the cooling of a rotating mirror in a L–G cavity containing an optical parametric amplifier (OPA). For different parametric phase and parametric gain of OPA, we discuss, in detail, the feasibility of achieving cooling of a rotating mirror. This paper is organized as follows. We give an analytic description of the optorotational system in Section 2, where the evolution equations of the system are analytically obtained. In Section 3, how to achieve the cooling of a rotating mirror by choosing appropriate physical parameters is discussed in detail. Finally, a conclusion of the results is summarized in Section 4.

## 2. Model and Methods

**Figure 1:**
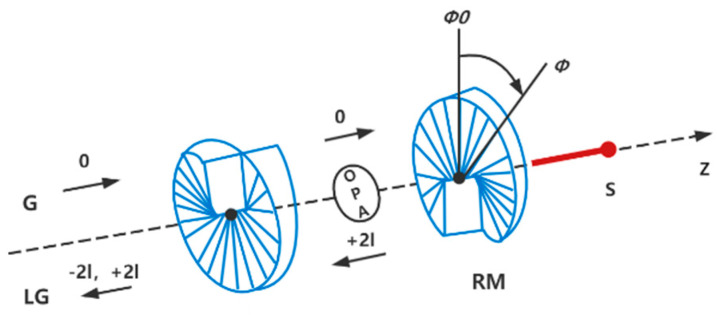
Sketch of a Laguerre–Gaussian cavity composed of two spiral phase elements: the right element represents a fixed mirror and the right one denotes a rotating mirror (MR) rotating around the cavity axis (z axis) which is suspended from a support S. The RM has an equilibrium position of $\phi_{0}=0$ and an angular displacement ϕ of rotation about the z axis. In addition, the cavity also contains a degenerate OPA. The L–G cavity is driven by an input Gaussian beam. When the Gaussian beam with topological charge 0 is incident upon the L–G cavity, the output field will be added a topological charge 2l from the mechanical torsional pendulum.

The system we consider is shown in Figure 1, which is a Laguerre–Gaussian optorotational system formed by two spiral phase elements: the left element represents a partially reflecting mirror and the right one stands for a perfectly reflecting mirror. The left mirror is fixed and the right mirror is rotating around the cavity axis. The right rotating mirror with the mass m and radius R can be seen as a mechanical torsional pendulum whose moment of inertia about the axis of rotation is defined as $I=mR^{2}$. The intrinsic damping rate and the angular frequency of the mechanical torsional pendulum are $D_{\phi }$ and $\omega_{\phi }$, respectively. When the Laguerre–Gaussian beams interact with a submicron particle or Bose–Einstein condensate, recent experiments demonstrate that the Laguerre–Gaussian beams can exert a torque on them [29,30]. Similarly, when the Laguerre–Gaussian optorotational system is driven by a fundamental-mode Gaussian beam, L–G beam may be generated by spiral phase elements. L–G beam can exert a torque on the right rotating mirror [31]. Here our optorotational system is driven by an incident Gaussian beam with the frequency $\omega_{L}$ and the positive amplitude $\varepsilon =\sqrt{2\kappa P/\left(\hbar \omega_{L}\right)}$, where P is the input laser power launched into the cavity. Note that κ is the cavity decay rate determined by κ=πc/(2FL), where F is the cavity finesse and L is the L–G cavity length when the cavity is not driven. The Gaussian beam with topological charge 0 which is incident upon the L–G cavity is reflected and the output light beam has added topological charge 2l from the rotating mirror. In other words, the coupling between the L–G cavity and the rotating mirror has arisen, because each reflected photon with initial charge 0 acquires angular momentum 2lℏ  from the rotating mirror [26]. The torque exerted by each photon in the L–G cavity to the mechanical torsional pendulum is $\hbar \xi_{\phi }=\hbar cl/L$, where $\xi_{\phi }=cl/L$ is the optorotational coupling parameter. Here, l represents orbital angular momentum. It is obvious that the exchange of orbital angular momentum between the photons and the mechanical torsional pendulum is the reason for the cooling of the mechanical torsional pendulum.

We write the Hamiltonian in a rotating frame with $\omega_{L}$ as the form

(1) $$H=\hbar \omega_{c}c^{†}c+\frac{1}{2}\left(\frac{L_{z}{}^{2}}{I}+I\omega_{\phi }{}^{2}\phi^{2}\right)-\hbar \xi_{\phi }c^{†}c\phi +i\hbar \varepsilon \left(c^{†}-c\right)+i\hbar G\left(e^{i\theta }c^{†}{}^{2}-e^{-i\theta }c^{2}\right)$$

where c and $c^{†}$ are the annihilation and creation operators of the cavity mode satisfying the communication relation $\left[c,c^{†}\right]=1$. Here, $\omega_{c}$ is the L–G cavity intrinsic frequency when the right mirror is stationary. $L_{z}$ is the angular momentum of the mechanical torsional pendulum about the cavity axis, ϕ is an angular displacement and they satisfy the communication relation $\left[\phi ,L_{z}\right]=i\hbar$. The first two terms on the right side of Equation (1) are the free energies of the cavity field and the mechanical torsional pendulum, respectively. The third term describes the interaction between the cavity and the mechanical torsional pendulum. The fourth term arises from the coupling of the cavity field and the input Gaussian field. The last term is the coupling between the OPA and the cavity field, G is the nonlinear gain of the OPA, and θ is the phase of the pump field driving the OPA.

The dynamics of the system can be described by the quantum Langevin equations

(2) $$\begin{array}{c} \dot{\phi }=\frac{L_{z}}{I},\;\\ \dot{L_{z}}=-I\omega_{\phi }{}^{2}\phi +\hbar \xi_{\phi }c^{†}c-\frac{D_{\phi }}{I}L_{z}+\varepsilon_{\phi }^{in},\;\\ \dot{c}=-i\left(\omega_{c}-\omega_{L}\right)+i\xi_{\phi }\phi c+2Ge^{i\theta }c^{†}-\kappa c+\sqrt{2\kappa }c_{in}+\varepsilon , \end{array}$$

where we have included the corresponding damping and noise terms. Here, $c_{in}$ refers to the input vacuum noise operator. Its mean value is zero and its fluctuations satisfy the correlation functions

(3) $$\begin{array}{c} \langle \delta c_{in}\left(t\right)\delta c_{in}{}^{†}\left(t^{\prime }\right)\rangle =\delta \left(t-t^{\prime }\right)\;,\;\\ \langle \delta c_{in}\left(t\right)\delta c_{in}\left(t^{\prime }\right)\rangle =0,\;\\ \langle \delta c_{in}{}^{†}\left(t\right)\delta c_{in}\left(t^{\prime }\right)\rangle =0. \end{array}$$

The force $\varepsilon_{\phi }^{in}$ is the Brownian noise operator which represents the mechanical noise that couples to the mechanical torsional pendulum from the thermal environment. It has zero mean value and the following correlation function when the temperature of the environment is T

(4) $$\langle \delta \varepsilon_{\phi }^{in}\left(t\right)\delta \varepsilon_{\phi }^{in}\left(t^{\prime }\right)\rangle =\frac{\hbar D_{\phi }}{2\pi }\int \omega e^{-i\omega \left(t-t^{\prime }\right)}\left[1+coth\left(\frac{\hbar \omega }{2k_{B}T}\right)\right]d\omega ,$$

where $k_{B}$ is the Boltzmann constant. Further we assume the cooling of the motion of the mechanical torsional pendulum is performed at high temperature and $k_{B}T\gg \hbar \omega$ holds, so we can simply Equation (4) by using $coth\left(\hbar \omega /2k_{B}T\right)=2k_{B}T/\hbar \omega$ approximately.

After a long period of evolution, the system will tend to a steady state. By setting Equation (2) equal to zero, we get the mean values of the system

(5) $$L_{z_{s}}=0,\;\phi_{s}=\frac{\hbar \xi_{\phi }{\left|c_{s}\right|}^{2}}{I\omega_{\phi }{}^{2}},\;c_{s}=\frac{\kappa -i\Delta +2Ge^{i\theta }}{\kappa^{2}+\Delta^{2}-4G^{2}}\varepsilon ,$$

where

(6) $$\Delta =\omega_{c}-\omega_{L}-\xi_{\phi }\phi_{s}$$

is the effective cavity detuning which is modified by $\xi_{\phi }\phi_{s}$ due to the optorotational interaction. The $\phi_{s}$ corresponds to the new equilibrium angular displacement of the mechanical torsional pendulum. $c_{s}$ is the amplitude of the cavity field at the steady state and ${\left|c_{s}\right|}^{2}$ is the number of photons in the L–G cavity.

In the following, we will focus on the fluctuations of the operators. We assume that the intracavity photon number ${\left|c_{s}\right|}^{2}$ is far greater than 1, which means the cavity field is driven by a strong light field. Under this assumption, we linearize Equation (2) to analyze the evolution of the system and examine the cooling of the mechanical torsional pendulum. Therefore, each operator in Equation (2) may be expressed as the sum of the steady-state mean value and a small fluctuation: $a=a_{s}+\delta a$, where a represents $\phi ,\;L_{z},\;c$, respectively. Then we obtain the evolution equation for the fluctuation operators

(7) $$\begin{array}{c} \delta \dot{\phi }=\frac{\delta L_{z}}{I},\;\\ \delta \dot{L_{z}}=-I\omega_{\phi }{}^{2}\delta \phi +\hbar \xi_{\phi }\left(c_{s}\delta c^{†}+c_{s}{}^{*}\delta c\right)-\frac{D_{\phi }}{I}\delta L_{z}+\varepsilon_{\phi }^{in},\;\\ \delta \dot{c}=-i\Delta \delta c+i\xi_{\phi }c_{s}\delta \phi +2Ge^{i\theta }\delta c^{†}-\kappa \delta c+\sqrt{2\kappa }\delta c_{in},\;\\ \delta {\dot{c}}^{†}=i\Delta \delta c^{†}-i\xi_{\phi }c_{s}{}^{*}\delta \phi +2Ge^{-i\theta }\delta c-\kappa \delta c^{†}+\sqrt{2\kappa }\delta c_{in}{}^{†}. \end{array}$$

Our work must be carried out when the system is stable. Thus, it is required to obtain the matrix form of Equation (7). Here we introduce the quadrature fluctuations in the cavity field $\delta x=\left(\delta c+\delta c^{†}\right)$, $\delta y=i\left(\delta c^{†}-\delta c\right)$ and the quadrature fluctuations in the input vacuum noise $\delta x_{in}=\left(\delta c_{in}+\delta c_{in}{}^{†}\right)$ and $\delta y_{in}=i\left(\delta c_{in}{}^{†}-\delta c_{in}\right)$, then Equation (7) can be written as

(8) $$\dot{u}\left(t\right)=Au\left(t\right)+n\left(t\right),\;$$

where $u{\left(t\right)}^{T}=\left(\delta \phi ,\delta L_{z},\delta x,\delta y\right)$ is the vector of the fluctuations and $n{\left(t\right)}^{T}=\left(0,\varepsilon_{\phi }^{in},\sqrt{2\kappa }\delta x_{in},\sqrt{2\kappa }\delta y_{in}\right)$ is the column vector of the input noise. Then the matrix A of Equation (8) is given by

(9) $$A=\left(\begin{array}{ccc} \begin{array}{c} 0\\ -I\omega_{\phi }{}^{2}\\ \begin{array}{c} i\xi_{\phi }\left(c_{s}-c_{s}{}^{*}\right)\\ \xi_{\phi }\left(c_{s}+c_{s}{}^{*}\right) \end{array} \end{array} & \begin{array}{c} \frac{1}{I}\\ -\frac{D_{\phi }}{I}\\ \begin{array}{c} 0\\ 0 \end{array} \end{array} & \begin{array}{cc} \begin{array}{c} 0\\ \hbar \xi_{\phi }\frac{c_{s}+c_{s}{}^{*}}{2}\\ \begin{array}{c} -\left(\kappa -2G\cos\theta \right)\\ \left(-\Delta +2G\sin\theta \right) \end{array} \end{array} & \begin{array}{c} 0\\ \hbar \xi_{\phi }\frac{c_{s}-c_{s}{}^{*}}{2i}\\ \begin{array}{c} \left(\Delta +2G\sin\theta \right)\\ -\left(\kappa +2G\cos\theta \right) \end{array} \end{array} \end{array} \end{array}\right).$$

The dynamic stability of the system we study depends on matrix A which allows us to correctly determine the range of some parameters. According to the Routh–Hurwitz criterion [32], the following three inequalities yield

(10) $$\begin{array}{c} 2\kappa \left(\kappa^{2}-4G^{2}+\Delta^{2}+2\kappa \frac{D_{\phi }}{I}\right)+\frac{D_{\phi }}{I}\left(2\kappa \frac{D_{\phi }}{I}+\omega_{\phi }{}^{2}\right)>0,\\ {\left(2\kappa +\frac{D_{\phi }}{I}\right)}^{2}\left(\frac{2\hbar \xi_{\phi }{}^{2}{\left|c_{s}\right|}^{2}\Delta }{I}+\frac{2\hbar \xi_{\phi }{}^{2}\left(c_{s}{}^{2}+c_{s}{{}^{*}}^{2}\right)G\sin\theta }{I}+\frac{2i\hbar \xi_{\phi }{}^{2}\left(c_{s}{}^{2}-c_{s}{{}^{*}}^{2}\right)G\cos\theta }{I}\right)+2\kappa \frac{D_{\phi }}{I}\left\{{\left(\kappa^{2}-4G^{2}+\Delta^{2}\right)}^{2}+\left(2\kappa \frac{D_{\phi }}{I}+{\left(\frac{D_{\phi }}{I}\right)}^{2}\right)\left(\kappa^{2}-4G^{2}+\Delta^{2}\right)+\omega_{\phi }{}^{2}\left[2\left(\kappa^{2}+4G^{2}-\Delta^{2}\right)+\omega_{\phi }{}^{2}+2\kappa \frac{D_{\phi }}{I}\right]\right\}>0,\;\\ \omega_{\phi }{}^{2}\left(\kappa^{2}-4G^{2}+\Delta^{2}\right)-\frac{2\hbar \xi_{\phi }{}^{2}{\left|c_{s}\right|}^{2}\Delta }{I}-\frac{2\hbar \xi_{\phi }{}^{2}\left(c_{s}{}^{2}+c_{s}{{}^{*}}^{2}\right)G\sin\theta }{I}-\frac{2i\hbar \xi_{\phi }{}^{2}\left(c_{s}{}^{2}-c_{s}{{}^{*}}^{2}\right)G\cos\theta }{I}>0. \end{array}$$

Note that the conditions (10) above are related to the parameters’ nonlinear gain G and phase θ of OPA.

The angular displacement fluctuation δϕ in the mechanical torsional pendulum can be obtained by solving the Fourier transform of Equation (7) in frequency space

(11) $$\delta \phi \left(\omega \right)=\frac{{\left(\kappa -i\omega \right)}^{2}+\Delta^{2}-4G^{2}}{d\left(\omega \right)}\varepsilon_{\phi }^{in}+\frac{\sqrt{2\kappa }\hbar \xi_{\phi }\left[2Ge^{-i\theta }c_{s}+\left(\kappa -i\omega -i\Delta \right)c_{s}{}^{*}\right]}{d\left(\omega \right)}\delta c_{in}+\frac{\sqrt{2\kappa }\hbar \xi_{\phi }\left[\left(\kappa -i\omega +i\Delta \right)c_{s}+2Ge^{i\theta }c_{s}{}^{*}\right]}{d\left(\omega \right)}\delta c_{in}{}^{†},$$

where

(12) $$d\left(\omega \right)=\left(I\omega_{\phi }{}^{2}-I\omega^{2}-iD_{\phi }\omega \right)\left[{\left(\kappa -i\omega \right)}^{2}+\Delta^{2}-4G^{2}\right]-2\hbar \xi_{\phi }{}^{2}\left[iGe^{-i\theta }c_{s}{}^{2}+{\left|c_{s}\right|}^{2}\Delta -iGe^{i\theta }c_{s}{{}^{*}}^{2}\right].$$

The first term in Equation (11) is from the thermal Langevin force, while the last two terms are from radiation torque. The angular displacement fluctuation in the mechanical torsional pendulum is determined by the total external forces that act on it. Generally, by taking Equation (11) as the form $\delta \phi \left(\omega \right)=\chi_{eff}\left(\omega \right)F_{t}\left(\omega \right)$, the effective susceptibility $\chi_{eff}\left(\omega \right)$ is defined which reflects the response of the mechanical torsional pendulum to the total external forces $F_{t}\left(\omega \right)$. Here, $\chi_{eff}\left(\omega \right)$ can be written as $\chi_{eff}\left(\omega \right)={\left(I\omega_{eff}{}^{2}-I\omega^{2}-iD_{eff}\omega \right)}^{-1}$ with the effective resonance frequency $\omega_{eff}\left(\omega \right)$ and the effective damping rate $D_{eff}\left(\omega \right)$ given by

(13) $$\omega_{eff}\left(\omega \right)={\left(\omega_{\phi }{}^{2}-\frac{2\hbar \xi_{\phi }{}^{2}}{I}\frac{\alpha \beta }{\gamma }\right)}^{\frac{1}{2}}$$

and

(14) $$D_{eff}\left(\omega \right)=D_{\phi }+\frac{2\hbar \xi_{\phi }{}^{2}\alpha \kappa }{\gamma }$$

where

(15) $$\begin{array}{c} \alpha =iGe^{-i\theta }c_{s}{}^{2}+{\left|c_{s}\right|}^{2}\Delta -iGe^{i\theta }c_{s}{{}^{*}}^{2}\\ \beta =\kappa^{2}-\omega^{2}+\Delta^{2}-4G^{2}\\ \gamma ={\left(\kappa^{2}-\omega^{2}+\Delta^{2}-4G^{2}\right)}^{2}+4\kappa^{2}\omega^{2}. \end{array}$$

Equation (13) shows that the angular frequency of the mechanical torsional pendulum makes change in the presence of $F_{t}\left(\omega \right)$, which is the so-called “optical spring effect”. Further, the different values of G and θ cause the modification of damping rate $D_{\phi }$ which leads to the cooling of the mechanical torsional pendulum. 

The spectrum of the angular displacement fluctuation in the mechanical torsional pendulum is given by

(16) $$S_{\phi }\left(\omega \right)=\frac{1}{4\pi }\int d\omega^{\prime }e^{-i\left(\omega +\omega^{\prime }\right)t}\langle \delta \phi \left(\omega \right)\delta \phi \left(\omega^{\prime }\right)+\delta \phi \left(\omega^{\prime }\right)\delta \phi (\omega )\rangle$$

By inserting Equations (3), (4) and (11) into Equation (16), we obtain

(17) $$S_{\phi }\left(\omega \right)=\frac{1}{{\left|d\left(\omega \right)\right|}^{2}}\left\{2\kappa \hbar^{2}\xi_{\phi }{}^{2}\left[\left(\kappa^{2}+\omega^{2}+\Delta^{2}+4G^{2}\right){\left|c_{s}\right|}^{2}+2Ge^{-i\theta }c_{s}{}^{2}\left(\kappa +i\Delta \right)+2Ge^{i\theta }c_{s}{{}^{*}}^{2}\left(\kappa -i\Delta \right)\right]+2k_{B}TD_{\phi }\left[{\left(\kappa^{2}-\omega^{2}+\Delta^{2}-4G^{2}\right)}^{2}+4\kappa^{2}\omega^{2}\right]\right\}$$

With the Fourier transform of $\delta \dot{\phi }=\delta L_{z}/I$ in Equation (7), we obtain the spectrum of the angular momentum fluctuation in the mechanical torsional pendulum

(18) $$S_{L_{z}}\left(\omega \right)=I^{2}\omega^{2}S_{\phi }\left(\omega \right).$$

In order to describe the limits for cooling, the final expressions for the two variances $\langle \delta \phi^{2}\rangle$ and $\langle \delta L_{z}{}^{2}\rangle$ can be obtained by performing the integrals $\frac{1}{2\pi }\int_{-\infty }^{+\infty }S_{\phi }\left(\omega \right)d\omega$ and $\frac{1}{2\pi }\int_{-\infty }^{+\infty }S_{L_{z}}\left(\omega \right)d\omega$. Furthermore, according to the law of thermal equilibrium, the effective temperature of the system can be defined as

(19) $$T_{eff}=\left[\frac{1}{2}I\omega_{\phi }{}^{2}\langle \delta \phi^{2}\rangle +\left(\langle \delta L_{z}{}^{2}\rangle /2I\right)\right]/k_{B}$$

We also introduce the parameter $r=\frac{1}{2}I\omega_{\phi }{}^{2}\langle \delta \phi^{2}\rangle /\left(\langle \delta L_{z}{}^{2}\rangle /2I\right)$ which can be regarded as the ratio of rotational kinetic energy to potential energy of the rotating mirror. According to the parameter, we can know the relative change in the angular displacement fluctuation and the angular momentum fluctuation in the mechanical torsional pendulum. When the rotating mirror is cooled close to the ground state, the value of parameter r approaches 1.

## 3. Results and Discussion

### 3.1. Effective Resonance Frequency and the Effective Damping Rate

The parameters we choose in the calculations are from Ref. [27]: the input Gaussian beam wavelength λ=810 nm, the input laser power P=50 mW, the cavity finesse $F=5\times 10^{3}$, the cavity length L=1 mm, the L–G cavity intrinsic frequency $\omega_{c}=2\pi \times 10^{14}\;\mathrm{Hz}$, the mass of the right mechanical torsional pendulum m=100 ng, the radius of the right mechanical torsional pendulum r=10 μm, the mechanical quality factor $Q_{\phi }=2\times 10^{6}$, and the frequency of the mechanical torsional pendulum $\omega_{\phi }=2\pi \times 10\;\mathrm{MHz}$. It has been proved that when $\Delta \;\approx \omega_{\phi }$, the cooling of the mechanical torsional pendulum can be optimally achieved in a dispersive optomechanical system [33].

**Figure 2:**
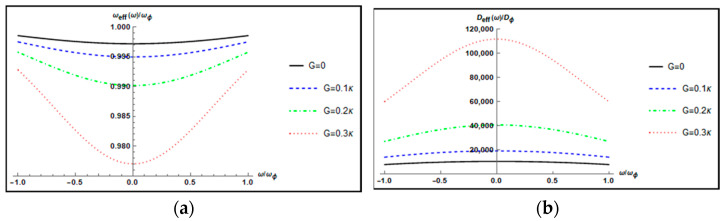
Plot of (**a**) the normalized effective resonance frequency $\omega_{eff}\left(\omega \right)/\omega_{\phi }$ and (**b**) the normalized effective damping rate $D_{eff}\left(\omega \right)/D_{\phi }$ of the mechanical torsional pendulum versus the normalized frequency $\omega /\omega_{\phi }$ for different parametric gains G=0, 0.1κ,0.2κ,0.3κ when $\Delta =\omega_{\phi }$, θ=0, and l=20.

In Figure 2, we plot the normalized effective resonance frequency $\omega_{eff}\left(\omega \right)/\omega_{\phi }$ and the normalized effective damping rate $D_{eff}\left(\omega \right)/D_{\phi }$ of mechanical torsional pendulum (the rotating mirror) as a function of the normalized frequency $\omega /\omega_{\phi }$ for different parametric gains G=0, 0.1κ, 0.2κ,0.3κ when $\Delta =\omega_{\phi }$, θ=0, and l=20. The normalized effective resonance frequency $\omega_{eff}\left(\omega \right)/\omega_{\phi }$ decreases gradually as parametric gain G increases and the decreasing trend is slow; that is, the influence of parametric gain G on the normalized effective resonance frequency is weak. However, the normalized effective damping rate $D_{eff}\left(\omega \right)/D_{\phi }$ is dramatically increased with increasing the parametric gain G. This is because increasing the parametric gain G leads to a larger photon number ${\left|c_{s}\right|}^{2}$ in the cavity, thereby generating a stronger optorotational coupling. The intracavity photon numbers ${\left|c_{s}\right|}^{2}$ are $2.996\times 10^{9}$, $4.134\times 10^{9}$, $6.305\times 10^{9}$, $1.105\times 10^{10}$ for G=0, 0.1κ,0.2κ,and 0.3κ, respectively.

**Figure 3:**
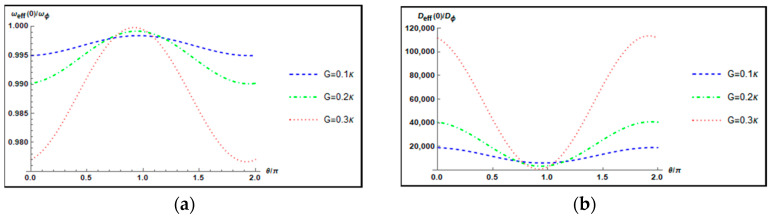
Plot of (**a**) the normalized effective resonance frequency $\omega_{eff}\left(0\right)/\omega_{\phi }$ and (**b**) the normalized effective damping rate $D_{eff}\left(0\right)/D_{\phi }$ of the mechanical torsional pendulum versus the parametric phase θ/π for different parametric gains G=0.1κ,0.2κ,0.3κ when $\Delta =\omega_{\phi }$, ω=0, and l=20.

Figure 3 plots the normalized effective resonance frequency $\omega_{eff}\left(0\right)/\omega_{\phi }$ and the normalized effective damping rate $D_{eff}\left(0\right)/D_{\phi }$ of the mechanical torsional pendulum as a function of the parametric phase θ/π for different parametric gains G=0.1κ,0.2κ,0.3κ when $\Delta =\omega_{\phi }$, ω=0, and l=20. Note that the maximum normalized effective damping rate $D_{eff}\left(0\right)/D_{\phi }$ is attained at θ=0. Therefore, in the following discussion, we always set θ=0.

### 3.2. Cooling of the Rotating Mirror with the OPA

In this section, we investigate the cooling effect of the mechanical torsional pendulum in the presence of the OPA.

#### 3.2.1. From Room Temperature *T* = 300 *K* to millikelvin Temperature

First, we assume that the initial temperature of the thermal environment is T=300*K*.

**Figure 4:**
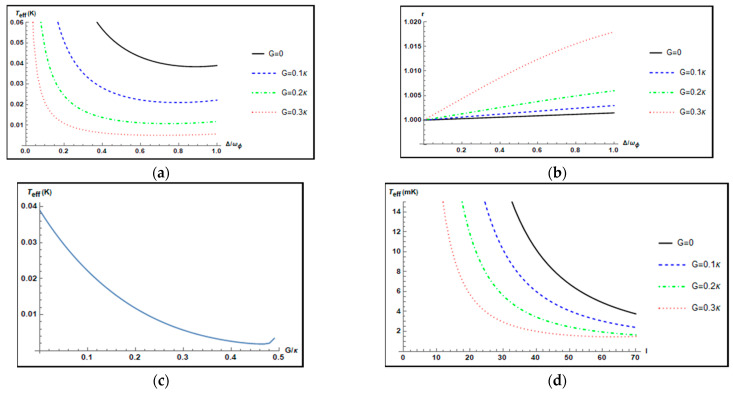
(**a**) Plot of the effective temperature $T_{eff}\left(K\right)$ of the mechanical torsional pendulum versus the normalized positive cavity detuning $\Delta /\omega_{\phi }$ for different parametric gains G=0, 0.1κ,0.2κ,0.3κ, θ=0, and l=20. (**b**) Plot of the parameter r versus the normalized positive cavity detuning $\Delta /\omega_{\phi }$ for different parametric gains G=0, 0.1κ,0.2κ,0.3κ when θ=0, and l=20. (**c**) Plot of the effective temperature $T_{eff}\left(K\right)$ of the mechanical torsional pendulum versus the parametric gain G of the OPA when $\Delta =\omega_{\phi }$, l=20, and θ=0. (**d**) Plot of the effective temperature $T_{eff}\left(\mathrm{mK}\right)$ of the mechanical torsional pendulum versus the orbital angular momentum l carried by the L–G cavity mode for different parametric gains G=0, 0.1κ,0.2κ,0.3κ when $\Delta =\omega_{\phi }$ and θ=0

In Figure 4a, we plot the effective temperature $T_{eff}\left(K\right)$ of the mechanical torsional pendulum as a function of the normalized positive cavity detuning $\Delta /\omega_{\phi }$. The black solid curve, the blue dashed curve, the green dot-dashed curve, and the red dotted curve are corresponding to different parametric gains G=0, 0.1κ, 0.2κ, 0.3κ, respectively, and other parameters θ=0 and l=20. When G=0, 0.1κ, 0.2κ, 0.3κ, it is found that the minimum values of the effective temperatures of the mechanical torsional pendulum are 0.0389K, 0.0221K, 0.0118K, and 0.0057K at $\Delta =\omega_{\phi }$, respectively. It is noted that the presence of the OPA with G=0.3κ and θ=0 can improve the cooling of the mechanical torsional pendulum by a factor of about 6 compared to that without the OPA (G=0). Figure 4b shows the parameter r against the normalized positive cavity detuning $\Delta /\omega_{\phi }$ for different parametric gains G=0, 0.1κ, 0.2κ, 0.3κ when θ=0, and l=20. It is noted that the parameter r is larger than 1, which indicates that the angular momentum fluctuations in the mechanical torsional pendulum are suppressed over the angular displacement fluctuations. Figure 4c plots the effective temperature $T_{eff}\left(K\right)$ of the mechanical torsional pendulum against the parametric gain G of the OPA when $\Delta =\omega_{\phi }$, l=20, and θ=0. We choose the parameter gain G of the OPA in a range of 0 to 0.495κ, which ensures that the system is stable. As Figure 4c shows, in the absence of the OPA (G=0), the effective temperature $T_{eff}$ of the mechanical torsional pendulum is 0.0389K. In the presence of the OPA, the effective temperature $T_{eff}$ of the mechanical torsional pendulum reaches the minimum value 0.0018K at G=0.46κ. Thus, the cooling of the mechanical torsional pendulum can be improved by a factor of about 21 using the OPA compared to the case without the OPA. However, as the parametric gain G continues to increase, the effective temperature $T_{eff}$ of the mechanical torsional pendulum rises. Therefore, it is necessary to choose the appropriate parametric gain G carefully in order to achieve the best cooling of the mechanical torsional pendulum. Figure 4d shows the effective temperature $T_{eff}\left(\mathrm{mK}\right)$ of the mechanical torsional pendulum against the orbital angular momentum l for different parametric gains of the OPA when $\Delta =\omega_{\phi }$ and θ=0. From Figure 4d, for a given value of the parametric gain G of the OPA, it is seen that the cooling of the mechanical torsional pendulum is improved with increasing the value of the orbital angular momentum l. The reason is that a larger value of orbital angular momentum l generates a stronger optorotational coupling. Without the OPA (G=0 and θ=0), the minimum value of the effective temperature $T_{eff}$ is 3.76 mK at l=70. With the OPA (G=0.3κ and θ=0), the minimum value of the effective temperature $T_{eff}$ is 1.50 mK at l=70. Thus, the cooling of the mechanical torsional pendulum can be improved by a factor of 2.5 with the help of the OPA.

#### 3.2.2. From Temperature *T* = 3 *K* to Submillikelvin Temperatures

We assume that the mechanical torsional pendulum couples to the thermal bath at temperature T=3K. The mechanical torsional pendulum can be cooled to a lower temperature when the OPA is introduced.

**Figure 5:**
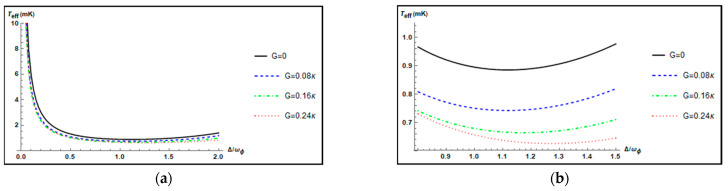
(**a**) Plot of the effective temperature $T_{eff}\left(K\right)$ of the mechanical torsional pendulum versus the normalized positive cavity detuning $\Delta /\omega_{\phi }$ for different parametric gains G=0, 0.08κ, 0.16κ, 0.24κ when l=20, T=3K and θ=0. (**b**) Enlargement of the curves of Figure 5a for the range of $\frac{\Delta }{\omega_{\phi }}\in \left[0.8,\;1.5\right]$.

In Figure 5, we plot the effective temperature $T_{eff}\left(K\right)$ of the mechanical torsional pendulum as a function of the normalized positive cavity detuning $\Delta /\omega_{\phi }$ for different parametric gains G=0, 0.08κ, 0.16κ, 0.24κ and other parameters l=20, T=3K, and θ=0. For a given value of the parametric gain G, it is seen that the effective temperature $T_{eff}\left(\mathrm{mK}\right)$ of the mechanical torsional pendulum is changed with the increase in the effective cavity detuning $\Delta /\omega_{\phi }$. We also find these four curves have their own minimum values for different parametric gains. In order to display the minimum value more clearly, the enlargement of the curves in Figure 5a for the range of $\frac{\Delta }{\omega_{\phi }}\in \left[0.8,\;1.5\right]$ is shown in Figure 5b. From Figure 5b, the black solid curve, the blue dashed curve, the green dot-dashed curve, and the red dotted curve correspond to G=0, 0.08κ,0.16κ,0.24κ, respectively. For each curve, we can find that the effective temperatures $T_{eff}\left(K\right)$ of the mechanical torsional pendulum have the minimum values $T_{eff}=0.8851\;\mathrm{mK},$ 0.7426 mK,  0.6643 mK, 0.6263 mK at $\Delta /\omega_{\phi }=1,\;\Delta /\omega_{\phi }=1.125,\;\Delta /\omega_{\phi }=1.175,\;\Delta /\omega_{\phi }=1.275$, respectively. We also calculate the effective mean phonon numbers corresponding to each curve at their respective lowest temperature. The corresponding effective mean phonon numbers of the mechanical torsional pendulum are about 1.344, 1.047, 0.884, and 0.805, respectively. It is shown that when G=0, the lowest temperature of the mechanical torsional pendulum is not at $\Delta =\omega_{\phi }$. As the parametric gain G in the OPA increases, the minimum value of the effective temperature $T_{eff}$ is obtained at a larger detuning$\;(\Delta /\omega_{\phi }>1)$.

## 4. Conclusions

In summary, we proposed a scheme where the cooling of a mechanical torsional pendulum in a Laguerre–Gaussian (L–G) cavity can be achieved in the presence of an OPA. For the parametric phase θ=0, which is different from the value in a Fabry–Perot cavity in Ref. [34] as well as a dissipative optomechanical system in Ref. [17], increasing the parametric gain G can lower the temperature of the mechanical torsional pendulum. The reason is that the increase in the parametric gain G changes the intracavity photon number to enhance the interaction between the cavity and the mechanical torsional pendulum, which makes the effective damping rate of the mechanical torsional pendulum increase significantly. Furthermore, the mechanical torsional pendulum can be cooled to sub-Kelvin temperatures from room temperature of 300K and to the ground state in a cryogenic environment by choosing the appropriate parametric gain G in an OPA and the cooling of a mechanical torsional pendulum with a larger value of the orbital angular momentum l can be improved by generating a stronger optorotational coupling. Finally, as the parametric gain G in the OPA increases, the minimum value of the effective temperature $T_{eff}$ is obtained at a larger detuning$\;(\Delta /\omega_{\phi }>1)$, which is not the same as cooling of a traditional optomechanical system [34]. Our model is an extension of the cavity optomechanical system. Our studies show that remarkable optorotational coupling of Laguerre–Gaussian spiral phase systems can provide a potential application in the determination of the orbital angular momentum of light fields and precision measurement.

Next, we discuss the feasibility of the experiment of our scheme. The generation of the L–G beam is crucial to the implementation of our scheme. Researchers can use spiral phase plates or spiral phase mirrors to produce the L–G beam [35]. We know that the spiral phase plate with a helical surface is an optical element. Photons reflected or transmitted by the spiral phase plate will obtain well-defined orbital angular momentum [36]. Therefore, using spiral phase plates to generate the L–G beam may be the most straightforward approach [35]. Current micro/nano-fabrication technology allows the spiral phase plate and mirror to be fabricated with high precision and low mass and the generation of the L–G beam with a topological charge value as high as 1000 has been demonstrated [37]. When a Gaussian mode beam passes through the spiral phase plate, it will be converted into L–G mode. Due to the transfer of orbital angular momentum, L–G beam can exert a torque on objects, including a submicron particle, a Bose–Einstein condensate, which has been realized in recent experiments [29,30]. The torques arising in the spiral phase elements as a result of the interaction with light have been extended to the Laguerre–Gaussian (L–G) optorotational system. In analogy with the cavity optomechanical system, the L–G optorotational system consists of two spiral phase elements acting as cavity mirrors (a concrete schematic diagram is shown in Figure 1), so the theoretical studies show that the Hamiltonian of the L–G optorotational system is similar to that of the cavity optomechanical system. Based on the torque from the L–G beam, trapping and cooling the rotational motion of a mirror, producing optomechanically induced transparency and realizing entanglement of a L–G cavity mode with a rotating mirror have been reported in the L–G optorotational system [24,26,27]. In addition, the parameter selections of our scheme are based on Ref. [27] and our scheme has feasibility in the experiment based on the research progress in the L–G optorotational system.

## Data Availability

Not applicable.
